# Next-generation sequencing provides an added value in determining drug resistance and viral tropism in Cameroonian HIV-1 vertically infected children

**DOI:** 10.1097/MD.0000000000010176

**Published:** 2018-03-30

**Authors:** Joseph Fokam, Maria C. Bellocchi, Daniele Armenia, Aubin J. Nanfack, Luca Carioti, Fabio Continenza, Desire Takou, Edith S. Temgoua, Charlotte Tangimpundu, Judith N. Torimiro, Paul N. Koki, Charles N. Fokunang, Giulia Cappelli, Alexis Ndjolo, Vittorio Colizzi, Francesca Ceccherini-Silberstein, Carlo-Federico Perno, Maria M. Santoro

**Affiliations:** a Chantal Biya International Reference Centre for research on HIV/AIDS Prevention and Management, Yaounde, Cameroon; b University of Rome Tor Vergata, Rome, Italy; c University of Yaounde I; d National HIV Drug Resistance Prevention and Surveillance Working Group, Yaounde, Cameroon; e New York University School of Medicine, New York, NY; f National Institute for Infectious Diseases Lazzaro Spallanzani-IRCCS, Rome, Italy; g Mother-Child Center, Chantal BIYA Foundation, Yaounde; h University of Bamenda, Bamenda, Cameroon; i National Research Council; j UNESCO Board of Multidisciplinary Biotechnology, Rome, Italy.

**Keywords:** children, coreceptor usage, HIV-1 drug resistance, next-generation sequencing, PMTCT, sanger sequencing

## Abstract

With limited and low-genetic barrier drugs used for the prevention of mother-to-child transmission (PMTCT) of HIV in sub-Saharan Africa, vertically transmitted HIV-1 drug-resistance (HIVDR) is concerning and might prompt optimal pediatric strategies.

The aim of this study was to ascertain HIVDR and viral-tropism in majority and minority populations among Cameroonian vertically infected children.

A comparative analysis among 18 HIV-infected children (7 from PMTCT-exposed mothers and 11 from mothers without PMTCT-exposure) was performed. HIVDR and HIV-1 co-receptor usage was evaluated by analyzing sequences obtained by both Sanger sequencing and ultra-deep 454-pyrosequencing (UDPS), set at 1% threshold.

Overall, median (interquartile range) age, viremia, and CD4 count were 6 (4–10) years, 5.5 (4.9–6.0) log_10_ copies/mL, and 526 (282–645) cells/mm^3^, respectively. All children had wild-type viruses through both Sanger sequencing and UDPS, except for 1 PMTCT-exposed infant harboring minority K103N (8.31%), born to a mother exposed to AZT+3TC+NVP. X4-tropic viruses were found in 5 of 15 (33.3%) children (including 2 cases detected only by UDPS). Rate of X4-tropic viruses was 0% (0/6) below 5 years (also as minority species), and became relatively high above 5 years (55.6% [5/9], *P* = .040. X4-tropic viruses were higher with CD4 ≤15% (4/9 [44.4%]) versus CD4 >15% (1/6 [16.7%], *P* = .580); similarly for CD4 ≤200 (3/4 [75%]) versus CD4 >200 (2/11 [18.2%] cells/mm^3^, *P* = .077.

NGS has the ability of excluding NRTI- and NNRTI-mutations as minority species in all but 1 children, thus supporting the safe use of these drug-classes in those without such mutations, henceforth sparing ritonavir-boosted protease inhibitors or integrase inhibitors for the few remaining cases. In children under five years, X4-tropic variants would be rare, suggesting vertical-transmission with CCR5-tropic viruses and possible maraviroc usage at younger ages.

## 1 Introduction

Despite increasing coverage (to about 61%) in prevention of mother-to-child transmission (PMTCT), human immunodeficiency virus type 1 (HIV-1) vertical-transmission remains consistent in sub-Saharan Africa (SSA).^[1]^ More so, although progress in PMTCT (from single-dose nevirapine [sd-NVP] to option-B+) has been reducing HIV-1 vertical-transmission, infected children stand at higher risks of HIV-1 drug resistance (HIVDR) to antiretrovirals administered pre-, per-, or post-partum.^[1,2]^ This is particularly true in SSA because of wide use of low genetic-barrier drugs, recurrent stock-outs, impaired-adherence, inadequate monitoring, HIV-1 diversity and, importantly, limited pediatric highly active antiretroviral therapy (HAART) options.^[3–5]^ All these factors lead to delayed detection of HAART failure and HIVDR accumulation even beyond 80%.^[6,7]^

As the footprint of long-term HAART depends largely on the effectiveness of first-line drugs in sustaining viral suppression, establishing adequacy between pediatric HAART and DR-mutations (DRMs) would be clinically relevant.^[7,8]^ In this line, we earlier reported low- and high-HIVDR, respectively, in naïve and HAART-failing children, with successful switch to second-line.^[9]^ From these observations, we postulated that minority DRMs in HAART-naïve children might grow-up through selective drug-pressure and populate plasma in a short-frame, herein justifying the rapidly emerging DRMs we observed at failure.^[9]^ Although not yet clinically endorsed, pediatric minority DRMs might be more concerning in the context of PMTCT, henceforth underscoring an unmet clinical need.^[10,11]^ Coupled to previous knowledge on the detection of DRMs by next-generation sequencing (NGS),^[12–14]^ we thus hypothesized that using NGS to assess DRMs in vertically infected HAART-naïve children would contribute in designing long-term HAART strategies for SSA-children.

Current pediatric HAART-regimens consist of lamivudine (3TC), abacavir (ABC), or zidovudine (AZT), associated to ritonavir-boosted lopinavir (LPV/r) or NVP. LPV/r is recommended to overcome PMTCT-resulting non-nucleoside reverse transcriptase inhibitor (NNRTI) resistance, whereas NVP matches with postnatal prophylaxis.^[15]^ As HAART would be reaching 1.5 million children by 2020, as high as 20% virological failure (VF) is expected, favored by high-viremia and poor adherence in children.^[15,16]^ Without optimal strategies, VF would quickly overcome HAART success, maintaining children vulnerable.^[17]^

Moreover, pediatric HAART options are limited in SSA, urging the quest for a wider therapeutic portfolio.^[3,8]^ Although not yet approved for under 16 years, the CCR5 antagonist—maraviroc—might represent a suitable antiretroviral alternative for children,^[18]^ pending proof-of-concept towards relevant pediatric clinical trials. Particularly, there are limited evidence on the potential effectiveness of maraviroc for SSA-children in PMTCT, initial-HAART and/or following treatment-failure.^[19–21]^ With rising concerns of minority variants on response to several classes of antiretrovirals,^[14]^ a genuine delineation of HIV-1 tropism, considering both minority and majority quasi-species,^[22,23]^ could rationalize maraviroc suitability for pediatric HAART-policies in SSA.

Based on these assumptions, we aimed to ascertain DRMs and HIV-1 co-receptor usage, in majority and minority viral populations, from children according to maternal PMTCT-exposure in a resource-limited setting (RLS).

## 2 Study design

### 2.1 Sampling and setting.

A comparative study was conducted in 2015 among 18 HIV-1 vertically infected Cameroonian children, all HAART-naïve, stratified according to maternal antiretroviral exposure during pregnancy: control-group (11 children from mothers without antiretroviral exposure) versus case-group (7 children from mothers exposed to reverse transcriptase inhibitors [RTIs]). For each child, a plasma sample was collected to perform both Sanger- and 454 ultra-deep pyrosequencing (UDPS).

### 2.2 Sanger sequencing.

Protease (PR)/RT Sanger sequencing was performed as previously described.^[24]^ Briefly, viral RNA was extracted from plasma using QIAamp Viral RNA minikit (Qiagen, Milan, Italy), following manufacturer's instructions. PR/RT-containing region was then reverse-transcribed and amplified using SuperScript One-Step for long templates reverse transcriptase polymerase chain reaction (RT-PCR) of Invitrogen kit (Foster City, CA), with an eventual second-round seminested PCR. Direct sequencing was then performed using 7 overlapping primers.

V3 loop Sanger sequencing was performed as previously described.^[25]^ Briefly, viral RNA containing the V3-loop region was reverse-transcribed and amplified using an RT/Taq mix, with an eventual second-round seminested PCR. Direct sequencing was then performed using 4 overlapping primers.

### 2.3 Amplification of PR/RT region for UDPS

Ten milliliters of viral RNA was reverse transcribed and amplified using 1-step RT-PCR system containing 25 μL reaction mix (2×), 8 μL MgSO4 (5 mmol/L), 2.8 μL H_2_O DNase RNase free, 1 μL forward primer (10 μmol/L), 1 μL reverse primer (10 μmol/L), 1 μL RNase Out (40 U/μL Invitrogen) and 1.2 μL RT/TAQ, for a final volume of 50 μL. RT-PCR conditions were the following: 1 cycle 50°C, 30 minutes; 1 cycle 94°C, 2 minutes; 40 cycles (94°C, 30 seconds; 51°C, 30 seconds; 68°C, 2 minutes); a final extension 68°C, 10 minutes. Forward and reverse primers were respectively 5’GACAGGCTAATTTTTTAGGG3’ (2075–2094 bps, gag) and 5’GATAAATTTGATATGTCCATTG3’ (3555–3576bps, pol). Nested-mid PCR was then performed with the Fast Start HiFi PCR system (Roche Diagnostics, Mannheim, Germany) using 5 pairs of barcoded-modified forward and reverse primers for each amplicon (Table 1). Based on band's size from eurosafe (Euroclone) agarose gel, 31.1 μL in water diluted cDNA was mixed per tube with 3.75 μL PCR buffer (10×), 0.75 μL dNTPs (12.5%), 0.75 μL forward primer (10 μmol/L), 0.75 μL primer (10 μmol/L) and 0.4 μL Taq, under the following conditions: 1 cycle 94°C, 3 minutes; 30 cycles (94°C, 30 seconds; amplicon annealing temperature, 30 seconds; 72°C, 35 seconds); a final extension 72°C, 7 minutes.

**Table 1:**
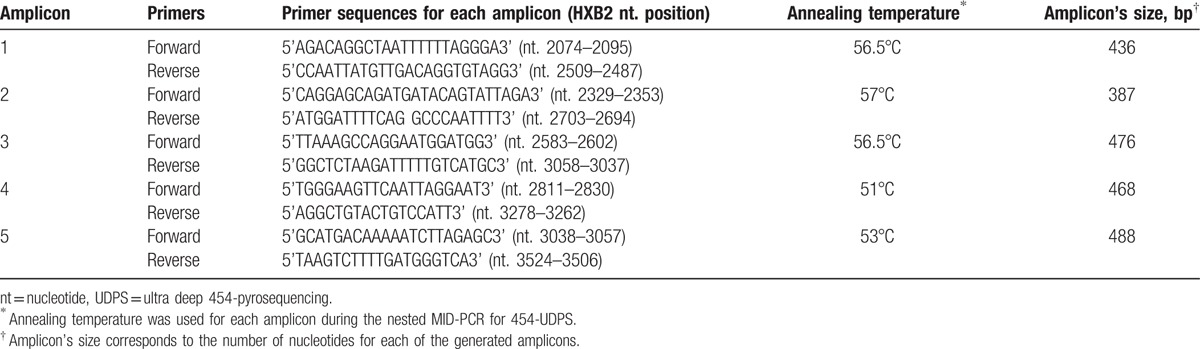
UDPS primers, annealing temperatures, and amplicon's size.

### 2.4 Amplification of V3 loop region for UDPS.

Ten microliters viral RNA were reverse transcribed with 1-step RT-PCR system using forward (gp120, 5’CCAATTCCCATACATTATTGT3’; 49–669 bps) and reverse (gp120, 5’CTTCTCCAATTGTCCCTCA3’; 1421–1439 bps) primers, under the following conditions: 1 cycle 50°C, 30 minutes; 1 cycle 94°C, 2 minutes; 35cycles (94°C, 30 seconds; 51°C, 30 seconds; 68°C, 1 minute and 30 seconds); a final extension 68°C, 10 minutes. A nested mid-PCR was then performed with the Fast Start HiFi PCR system (Roche Diagnostics, Mannheim, Germany) as previously described.^[26]^

### 2.5 Amplicon purification and UDPS reaction.

PR/RT PCR products (5 fragments of 436, 387, 476, 468 and 488 bps) and V3 loop (one fragment of 367 bps) were purified using Agencourt AMPure PCR purification beads (Beckman Coulter, Brea, CA) and quantified with Quant-iT PicoGreen double-stranded DNA assay kit (Life Technologies, Eugene, OR) on a GloMax multidetection system (Promega, Madison, WI).

Pooled purified PCR products were clonally amplified by emulsion PCR and pyro-sequenced on the 454 GS junior platform (Roche Applied Science, Mannheimer Germany) as previously described.^[26]^ Phylogenetic analyses excluded any possible sample contamination (data not shown).

### 2.6 Bioinformatics analyses of PR/RT and V3 sequences.

The entire PR (amino acid position: 1–99), RT (1–251) and the entire V3 loop (1–35) sequences obtained after 454-pyrosequencing were de-multiplexed and then quantified using the SFF tool Roche. Using a home-made Perl script and SHORAH package 0.5.1, sequences were filtered and corrected for homopolymeric region-associated errors and aligned against HIV-1 consensus B. Final alignments were manually checked for insertion or deletion in homopolymeric regions that could result in a frame shift. Nucleotidic/aminoacidic variants were evaluated and quantified by a home-made pearl script, and sequences were considered reliable when showed an intra-patient frequency ≥1% in both forward and reverse strands.

### 2.7 HIV drug resistance interpretation and viral-tropism determination.

PR/RT DRMs and HIV-1 co-receptor usage were interpreted using Stanford HIVdb list (updated March 9, 2015, available at http://hivdb.stanford.edu/pages/download/resistanceMutations_handout.pdf) and geno2pheno.v2.5 (http://coreceptor.geno2pheno.org/), respectively. Using a quantitative interpretation, viruses were considered CXCR4-tropic (X4-variants) by UDPS when ≥2% viral species had a false-positive rate (FPR) ≤3.5%,^[27]^ or by Sanger sequencing when FPR was ≤10%, describing the probability of classifying an R5-virus falsely as an X4-variant.^[25]^

### 2.8 HIV-1 subtyping

Subtyping was performed through phylogenetic analysis, by aligning all PR/RT Sanger-sequences in Bio-Edit compared to reference sequences of HIV-1 subtypes and circulating recombinant forms (CRFs) available at http://www.hiv.lanl.gov as previously described.^[28]^

### 2.9 Statistical analysis

HIV-1 DRMs and coreceptor usage were compared between the two PMTCT-groups. Coreceptor results by Sanger sequencing and UDPS were considered concordant if viral-tropism was identical from both sequencing technologies. Viral-tropism was explored according to age and CD4 count.

All statistical analyses were performed using the statistical open source environment R.v.3.1.1. *P* values <.05 were considered statistically significant.

### 2.10 Ethical considerations.

Ethical clearance was obtained from the Cameroon National Ethics Committee (*Ref. N°034/NEC/SE*), proxy-informed consent was provided, unique identifiers were used for privacy and confidentiality, and a material transfer agreement was established.

## 3 Results

### 3.1 Characteristics of children analyzed.

Overall, median (interquartile range [IQR]) age, viremia, and CD4 count were 6 (4–10) years, 5.5 (4.9–6.0) log_10_ copies/mL, and 526 (282–645) cells/mm^3^, respectively, without any significant difference between the 2 groups (data not shown). In the control, neither children nor their mothers had any antiretroviral exposure. Antiretroviral history of children belonging to the case-group, considered at higher risk of HIVDR, is described in Table 2.

**Table 2:**
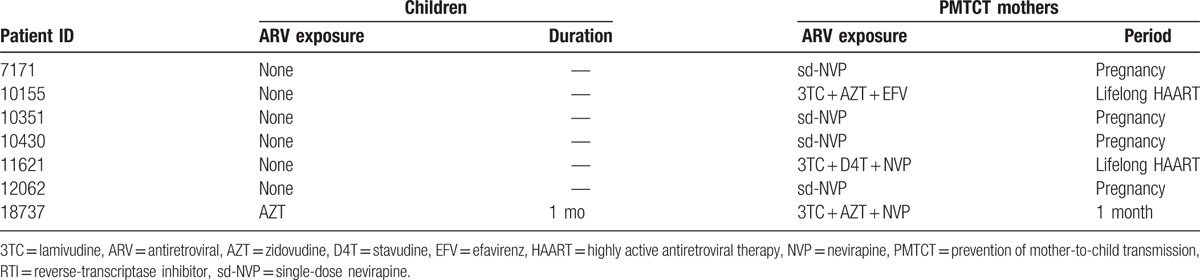
Antiretroviral history of children with PMTCT exposure.

### 3.2 HIV-1 subtype distribution.

HIV-1 subtyping revealed 50% CRF02_AG (9/18), 33.3% F (6/18), 11.1% CRF01_AE (2/18), and 5.6% CRF11.cpx (1/18).

### 3.3 HIV-1 drug resistance in the children analyzed.

PR/RT sequences were successfully obtained both through Sanger sequencing and UDPS for 17/18 children. The median UDPS coverage was of 1642 (IQR: 1269–5193) reads. In the entire covered PR/RT regions, the 2 sequencing technologies showed total concordance in variants detection, and all UDPS variants with frequencies <20% were not detected by Sanger sequencing (Table 3).

**Table 3:**
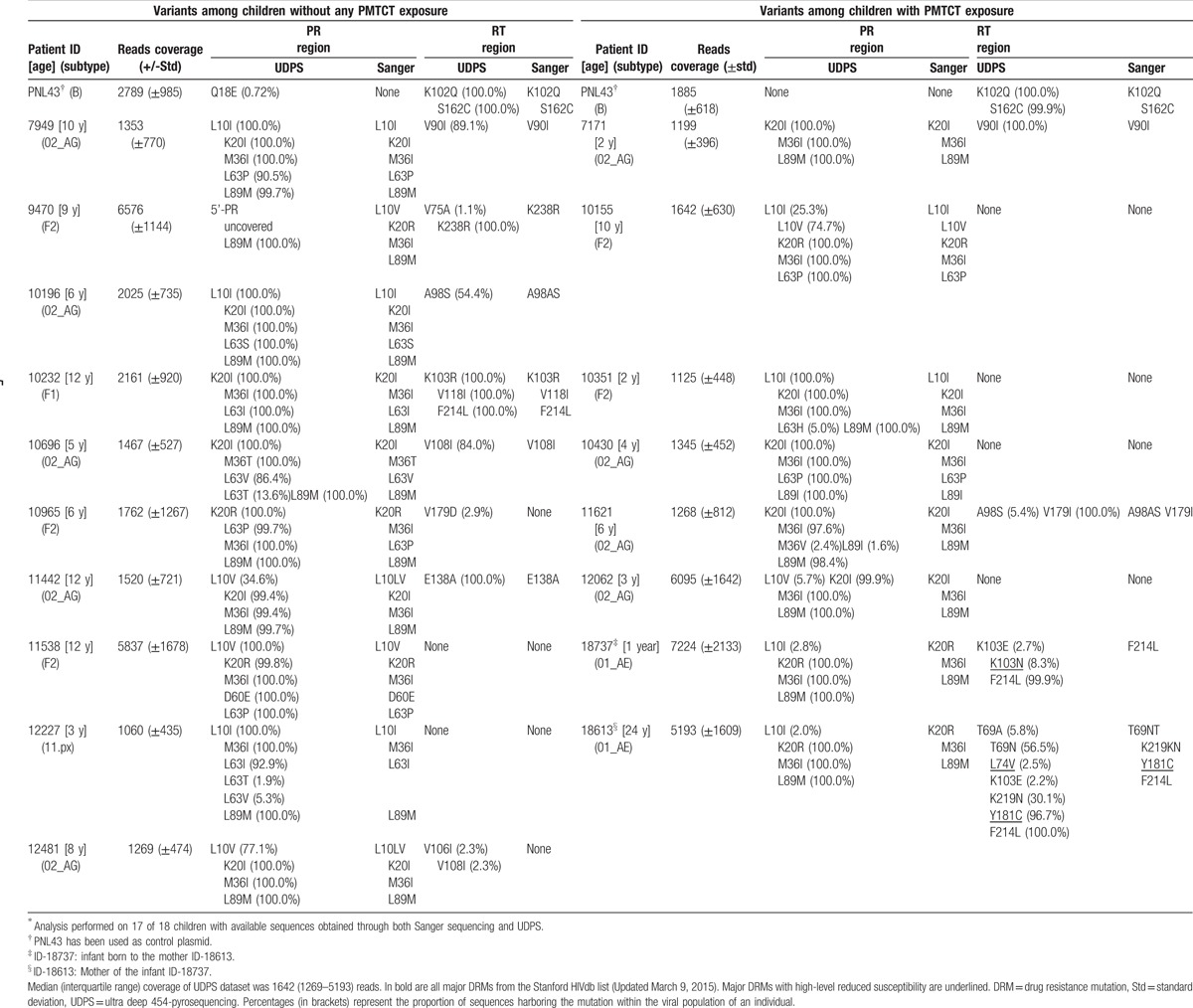
HIV-1 DRMs according to sequencing technologies: 454 UDPS versus Sanger sequencing^∗^.

By using Sanger sequencing, all 17 children had a wild type virus. Only E138A (5.9%), an accessory polymorphism weakly selected under etravirine (ETR) and rilpivirine (RPV), was found in a child aged 8 years from the control group.

By using UDPS, 1 (aged 1 year) of 7 children (14.3%) from the case-group harbored viruses with K103N (8.3% prevalence; mutational load: 190,567 copies/mL), a nonpolymorphic mutation causing high-level resistance to NVP and efavirenz (EFV). This infant was born from an RTI-treated mother (AZT + 3TC + NVP). Thus, Sanger sequencing and UDPS were performed also for the mother (ID-18613). UDPS revealed a virus harboring 2 major DRMs: L74 V at minority-level (2.5%), causing high- and intermediate-level resistance respectively to didanosine and to ABC; Y181C at population-level (96.7%), causing high- and intermediate-level resistance respectively to NVP and to EFV, ETR, and RPV (Table 3). No minority DRMs were found in any of all other 6 children from the case-group.

In the control-group, UDPS detected V179D at minority-level (2.9%), a polymorphic accessory mutation selected under EFV, in a child aged 6 years (Table 3).

Other variants, found even at RTI-associated drug resistance positions, were with minimal or no effect on drug susceptibility or virological response. Of note, in either group, no major DRMs to ritonavir-boosted protease inhibitors (PI/r) were found by both Sanger sequencing and UDPS.

### 3.4 HIV-1 co-receptor tropism in the children analyzed.

V3 loop sequencing was successful by both Sanger sequencing and UDPS for 15 of 18 children and the mother ID-18613, with an overall viral-tropism concordance of 87.5% (14/16) between Sanger sequencing and UDPS (Table 4).

**Table 4:**
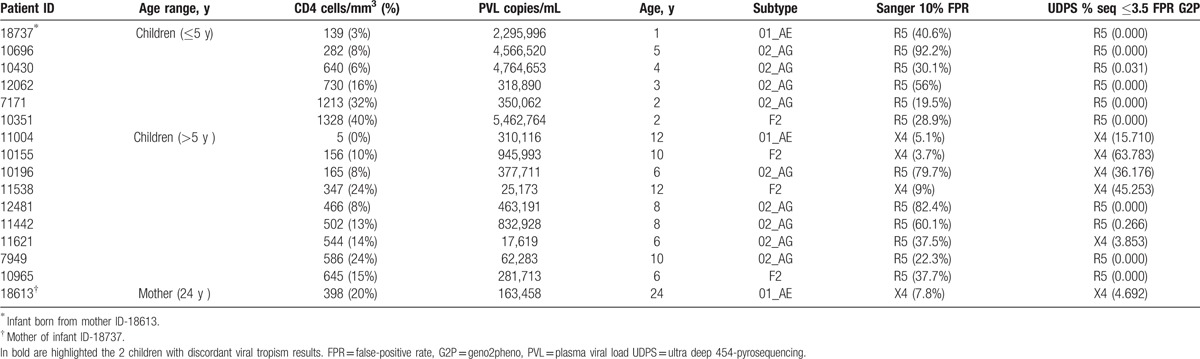
Viral-tropism according to sequencing technologies.

X4-tropic viruses were found in 5 of 15 (33.3%) children (including 2 cases detected only by UDPS), all aged above 5 years. Specifically, in 1 child (ID-11621) UDPS provided an added value in tropism-determination compared to Sanger sequencing. Indeed, a clinically relevant quantity of minority X4-tropic variants (frequency: 3.9%) was detected by UDPS in this child (low mutational load: 679 copies/ml). In another child (ID-10196), despite an R5-tropism (FPR = 79.7%) determined by Sanger sequencing, a discordant tropism was observed through UDPS with a high percentage of X4-tropic variants (36.2%, high mutational load: 136,641 copies/mL), because of insertions detected only at minority levels.

Of relevance, the rate of X4-tropic viruses was 0% (0/6) among children under 5 years (also as minority species at 1% the threshold), and became significantly higher as from 5 years and above (55.6% [5/9], *P* = .040). As expected, X4-tropic viruses were higher with CD4 ≤15% (4/9 [44.4%]) versus CD4 >15% (1/6 [16.7%], *P* = .580); similarly for CD4 ≤200 (3/4 [75%]) versus CD4 >200 (2/11 [18.2%] cells/mm^3^, *P* = .077). No statistical difference was found in X4-variants between the 2 PMTCT-groups: 2 of 7 (28.6%) case group versus 3 of 8 (37.5%) control group, *P* = 1.000.

## 4 Discussion

Sustaining HAART success remains challenging for children in a long term, especially in a context where adherence and drug options are limited.^[2,4,5]^ Thus, novel strategies are required to limit the spread of preventable HIVDR and provide alternative therapeutics with utmost potency for SSA children.^[29,30]^

In this high CRF02_AG-infected population,^[6,9,31,32]^ HAART-naïve children appeared with wild-type viruses at population-levels, confirming the low-level of HIVDR previously reported of this target-group.^[9,33]^ Interestingly, a vertically transmitted minority DRM (K103N), known to be associated with resistance to NNRTIs used both for PMTCT and first-line HAART in SSA, was found in a PMTCT-exposed infant, thus suggesting NNRTI-sparing regimens for such children.^[7,30,34]^ Discrepancy in DRMs between mother and infant would be due to sample collection later after delivery (at the moment of infant HIV diagnosis), with possible selection following prophylaxis/breastfeeding; as previously reported in similar RLS (Kyela, Tanzania).^[35]^ This infant (aged 1 year), compared to the median age of the study population (6 years), suggests that circulating DRMs might have fade-up with increasing age.^[7,33]^ NNRTI mutations (E138A and V179D), found in children without PMTCT-exposure, are known as polymorphisms with little or no effect on drug susceptibility or virological response.^[29]^ The ability of NGS in excluding minority RTI-mutations (in all but one children) supports the safe use of NNRTIs/NRTIs in those without such mutations, thus sparing from inappropriate switch to PI/r- or integrase inhibitor-containing regimens.^[7,8,13,17,33–35]^

Coreceptor usage in these children provides a clue for clinical application. Indeed, X4-variants appeared to be associated with older ages and lower CD4 cells, suggesting limited vertical transmission by CXCR4-tropic viruses, and later appearance of X4-variants with chronicity, immunological impairment,^[36,37]^ as well as a baseline FPR <60 as previously demonstrated.^[38,39]^ Further investigations might help in establishing novel public health strategies for an eventual usage of maraviroc in children.^[18,40]^ As current PMTCT-practice might not be an independent factor for viral-tropism (i.e., similar distribution in X4-variants irrespective of PMTCT-history), CCR5-antagonist (maraviroc) could be a useful therapeutic weapon for pediatric HAART.^[15,18,40]^

Of the two children showing discordant results between the two sequencing techniques, the added value of UDPS in detecting X4-tropic minority variants is in accordance with previous reports.^[13,39]^ Interestingly, by detecting minority insertions associated with a complete discrepant result on Sanger sequencing, UDPS appears very useful in validating tropism determination for non-B subtypes.^[41]^

Therefore, UDPS might provide additional information in detecting DRMs and viral-tropism, confirming the added value of this technology for both clinical diagnostics and management of non-B HIV-infected children.^[21,22,41]^

In spite of this added value of UDPS, implementing NGS is more challenging in RLS (costs, technical complexity, maintenance), suggesting the need for simpler and affordable approaches integrating minority variants (point-of-care or pragmatic sequencing).^[42,43]^

A potential study limitation could be the relatively small sample size, which makes the study probability relatively large. Also, in the PMTCT-exposed group, only 3 of 7 were exposed to triple ART, calling for subsequent investigations with scale-up of option B+. Moreover, HIV-1 variants were investigated only in plasma compartment, suggesting the need for exploring HIV variability in several compartments (cellular reservoirs, central nervous systems, among others) and the impact on treatment and monitoring strategies in SSA.^[12,13,44–46]^ This study therefore provides relevant data to be used as base for further/enlarged studies.

In a nutshell, NGS could help in identifying PMTCT-exposed children harboring minority NNRTI-DRMs, therefore serving for a timely switch of treatment and limiting failure rate. NGS also reveals a possible absence of X4-variants among children below 5 years, thus suggesting possible public health approaches using maraviroc. These preliminary evidences, generated on a small sample of mainly CRF02_AG-infected individuals, merit further investigations for improved pediatric-HAART strategies in RLS.

## Author contributions

**Conceptualization:** A. Nanfack, C-F. Perno, C. Fokunang, C. Tangimpundu, D. Armenia, D. Takou, E. Temgoua, F. Ceccherini-Silberstein, G. Cappelli, J. Fokam, M-M. Santoro, P. Koki, V. Colizzi.

**Data curation:** D. Armenia, F. Ceccherini-Silberstein, J. Fokam, L. Carioti, M. Bellocchi, M-M. Santoro.

**Formal analysis:** D. Armenia, F. Ceccherini-Silberstein, J. Fokam, L. Carioti, M. Bellocchi, M-M. Santoro.

**Funding acquisition:** C-F. Perno, J. Fokam, V. Colizzi.

**Investigation:** A. Ndjolo, A. Nanfack, C-F. Perno, C. Fokunang, C. Tangimpundu, D. Takou, E. Temgoua, F. Ceccherini-Silberstein, G. Cappelli, J. Fokam, J. Torimiro, M-M. Santoro, P. Koki, V. Colizzi.

**Methodology:** D. Armenia, D. Takou, F. Continenza, F. Ceccherini-Silberstein, J. Fokam, L. Carioti, M. Bellocchi, M-M. Santoro.

**Project administration:** A. Ndjolo, C-F. Perno, C. Fokunang, F. Ceccherini-Silberstein, G. Cappelli, J. Fokam, J. Torimiro, P. Koki, V. Colizzi.

**Resources:** A. Ndjolo, C-F. Perno, J. Fokam, V. Colizzi.

**Software:** D. Armenia, D. Takou, F. Continenza.

**Supervision:** A. Ndjolo, C-F. Perno, C. Fokunang, F. Ceccherini-Silberstein, G. Cappelli, M-M. Santoro, P. Koki, V. Colizzi.

**Validation:** A. Ndjolo, C-F. Perno, C. Fokunang, C. Tangimpundu, D. Armenia, E. Temgoua, F. Continenza, F. Ceccherini-Silberstein, G. Cappelli, J. Fokam, J. Torimiro, L. Carioti, M. Bellocchi, M-M. Santoro, P. Koki, V. Colizzi.

**Visualization:** A. Nanfack, C-F. Perno, C. Tangimpundu, D. Takou, E. Temgoua, F. Continenza, J. Fokam, M-M. Santoro.

**Writing – original draft:** C-F. Perno, F. Ceccherini-Silberstein, J. Fokam, M-M. Santoro.

**Writing – review & editing:** A. Ndjolo, A. Nanfack, C. Fokunang, C. Tangimpundu, D. Armenia, D. Takou, E. Temgoua, F. Continenza, G. Cappelli, J. Torimiro, L. Carioti, M. Bellocchi, P. Koki, V. Colizzi.

## Acknowledgements

We are appreciative to our institutional staff that participated locally in the enrolment and in sample processing. We thank Domenico Di Carlo for statistical analyses.
